# Statistics of Knots, Geometry of Conformations, and Evolution of Proteins

**DOI:** 10.1371/journal.pcbi.0020045

**Published:** 2006-05-19

**Authors:** Rhonald C Lua, Alexander Y Grosberg

**Affiliations:** Department of Physics, University of Minnesota, Minneapolis, Minnesota, United States of America; Harvard University, United States of America

## Abstract

Like shoelaces, the backbones of proteins may get entangled and form knots. However, only a few knots in native proteins have been identified so far. To more quantitatively assess the rarity of knots in proteins, we make an explicit comparison between the knotting probabilities in native proteins and in random compact loops. We identify knots in proteins statistically, applying the mathematics of knot invariants to the loops obtained by complementing the protein backbone with an ensemble of random closures, and assigning a certain knot type to a given protein if and only if this knot dominates the closure statistics (which tells us that the knot is determined by the protein and not by a particular method of closure). We also examine the local fractal or geometrical properties of proteins via computational measurements of the end-to-end distance and the degree of interpenetration of its subchains. Although we did identify some rather complex knots, we show that native conformations of proteins have statistically fewer knots than random compact loops, and that the local geometrical properties, such as the crumpled character of the conformations at a certain range of scales, are consistent with the rarity of knots. From these, we may conclude that the known “protein universe” (set of native conformations) avoids knots. However, the precise reason for this is unknown—for instance, if knots were removed by evolution due to their unfavorable effect on protein folding or function or due to some other unidentified property of protein evolution.

## Synopsis

Proteins in their native state are compact structures consisting of long chains of amino-acid residues. As such, a protein should be likely to get entangled or tie into a complex knot. However, researchers have found only a handful of complex knots in native proteins. Lua and Grosberg make what they believe to be the first quantitative study of the statistics of knots in proteins. Although they have found some rather complex knots, including one knot with five crossings in a modest size protein of only 229 amino acids (ubiquitin hydrolase), comparison of the knot abundance in proteins and in compact random strings on a lattice indicates extreme nonrandomness of protein conformations in this respect. They also study the statistics of the geometrical behaviour of parts of protein chains. They find that these parts, on the scale of about 20–30 residues, have a strong nonrandom tendency to crumple back on themselves, and that the segregration of the parts on this scale is also far in excess of random, while on a larger scale the geometry of conformations is statistically close to random. These geometrical features are consistent with the statistical rarity of knots. From these, the authors conclude that the “protein universe” avoids knots.

## Introduction

Few proteins with knotted native state conformations have been identified so far [1–7]. Therefore, it is tempting to conclude that the entire issue of knots is basically irrelevant for proteins. The goal of this paper is twofold:

First, we intend to give a more solid statistical justification to the statement that knots are rare in native proteins, namely, that they are significantly less frequent than random. To judge that the dearth of knotted proteins is indeed unusual, one has to possess statistics about knots in random compact loops (or globules) with which to compare. Simple models of such compact loops are ones that reside on a cubic lattice [8–10]. However, we feel that such comparisons have not been made explicitly and systematically in previous works. We offer such a comparative study here.

Second, we want to show that the perceived attitude about the rarity of knots, which is that the whole issue of knots is irrelevant, might not be correct. We propose that knots were likely to have been selected away, and, therefore, their absence may provide an important clue on the nature of the ensemble of protein conformations (“protein universe,” in the terminology of [11]).

Because we are interested in the topological properties of the protein molecule backbone, it suffices to represent each amino-acid residue with a single atom, which we choose to be the *α*-carbon atom, and to represent the continuous chain by straight segments connecting these *α*-carbon atoms.

In general, knots in any string are meaningfully defined as long as the ends either connect to each other, as for example in a plasmid DNA, or effectively go outward toward infinity, as in tightened shoelaces. Proteins are like neither of these two cases. If we simply connect the chain terminals with a straight line, or connect each terminal to infinity by the continuation of this straight line, or come up with any other specific way to complete the loop for every protein, we leave open the possibility that whatever knot we observe, a trivial knot or otherwise, is in fact due at least as much to the completing segments as to the protein itself. To overcome this problem, we use a statistical procedure, which shares some ideas with the random bridging of terminals used in [1] and with the study of the “spectrum” of knots in linear random walks made in [12]. Once a closed loop is obtained from a protein chain, we use knot invariants to identify the knot type. The major idea is to use a large ensemble of different random loop completions to see what kind of knot ensemble results. If a certain knot results from the majority of different loop completions, it is safe to conclude that this particular knot is an inherent property of the protein itself. (When this manuscript was being prepared for submission, [13] became available to us. In it, the methods in [12] were applied to the eight knotted protein segments initially studied in [3].)

We generate compact self-avoiding loops on a lattice using methods described in [8–10]. This model neglects the fine, atomic details represented by the sterics and energetics of amino-acid residues or by the Ramachandran plot. Because the knots in proteins are going to be determined at scales much larger than ten residues, we argue that these local atomic details are not going to be relevant.

To be able to compare the knotting probabilities of proteins with those of compact loops on a lattice, we have to determine what length of protein is comparable to a given length *N* of a compact lattice loop (or equivalently we have to determine the relevant persistence length). To this end, we examine the behaviour of subchains, which are short pieces of the whole protein chain or loop. Specifically, we generate data for the mean square end-to-end distance of subchains as a function of the length of the subchains. For short subchains, the secondary structures in proteins are apparent, and one obtains a scaling of subchain size reminiscent of stretched-out configurations. For longer subchains, the mean square end-to-end distance versus length of protein subchains approaches Gaussian or random walk behavior, an observation noted in [14]. The subchains of compact lattice loops likewise behave like a Gaussian, as was already shown in the study of large compact loops on a lattice in [10]. The results for the knotting probabilities in a lattice are also described in [10].

There is also a regime in the scaling of protein subchains reminiscent of chains confined to a small volume. For example, subchains in compact lattice loops experience a saturation or plateau in their end-to-end distance as the subchain size approaches that of the whole conformation. For protein subchains between about 15–40 residues long (equivalent to a few secondary structures in length), the scaling of the end-to-end distance also saturates, indicating a significant degree of compaction at scales less than the size of the protein chain. This feature is fairly robust and seems to take place at the same subchain lengths for most proteins, regardless of size or number of domains. The saturation has been identified as a turning back, on average, of the chain direction [14]. This saturation in the end-to-end distance is also consistent with the observed universality of closed-loop elements in globular proteins [15–17]. The origin of this feature could be traced to the tendency, as clearly seen in protein motifs or folds, of secondary structures adjacent along the chain to fold back on themselves [5,17,18]. (Some motifs displaying such regular arrangements have names such as “hairpin,” “meander,” “Rossmann fold,” “Greek key,” etc.)

Such tendency of subchains to be more compact on average has been shown to correlate with the absence of nontrivial (complex) knots in compact lattice loops [10]. For proteins, the saturation of the subchains at the scale of about 15–40 residues leads to a degree of interpenetration of the subchains, quantified below, which is less than that of compact lattice loops of size 6 × 6 × 6.

## Results

### Knotting Probabilities for Proteins Compared with Lattice Loops

The result presented in Figure 1 implies that the way to compare the knotting probability for proteins and lattice loops is to look at the correspondence between the number of monomers in the lattice system and the contour length of protein chains divided by 3.8 *Å,* which happens to be fairly close to the number of amino-acid residues.

**Figure 1:**
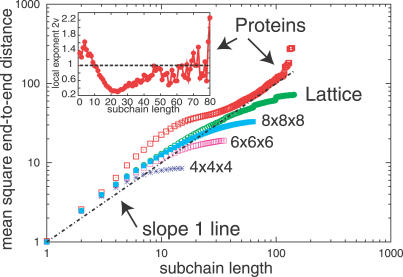
Data for the Mean Square End-to-End Distance of Subchains of Proteins (Squares) and Compact Lattice Loops (Circles) Plotted against the Subchain Length in a *log–log* Scale The mean square end-to-end distance of subchains for compact lattice loops of sizes 4 × 4 × 4, 6 × 6 × 6, and 8 × 8 × 8 also are shown to illustrate saturation at different loop sizes. For each chain of length *N*, subchains of length up to *N* ^2/3^ contribute to the average. The dashed line corresponds to a random walk behavior 〈*R* ^2^(*ℓ*)〉 = *ℓ*. The mean square end-to-end distance in Å^2^ for proteins has been divided by the factor (3.8)^2^. The data for proteins is similar to that in Figure 2 of [14]. (In that work, the end-to-end distance instead of the square of the end-to-end distance is plotted). The inset at the upper left shows the local scaling exponent 2*ν*, where 〈*R* ^2^(*ℓ*)〉 ∼ *ℓ* ^2ν^, plotted against subchain length (up to 80 residues) for proteins. 2*ν* was calculated from two adjacent protein data points at *ℓ*_1_ and *ℓ*_2_ via 2ν = log [〈*R* ^2^(*ℓ*_2_)〉/〈*R* ^2^(*ℓ*_1_)〉]/log(*ℓ*_2_ */ℓ*_1_). The horizontal dashed line in the inset represents the exponent 2ν = 1.

**Figure 2:**
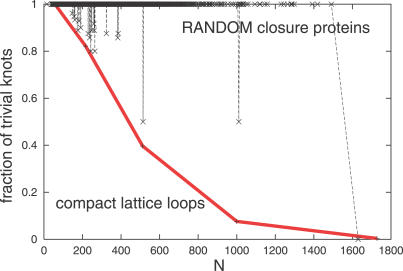
Fraction of Protein Chains at a Given Length with a Trivial Knot (0_1_) in the RANDOM Method, Plotted against the Length or Number of Residues Adjacent points are connected by dashed lines. The data for the trivial knotting probability of compact lattice loops (from 4 × 4 × 4 to 12 × 12 × 12) is included, shown connected by thick lines.

The probabilities of trivial knots (“no knots”) for proteins and compact lattice loops are plotted together and presented in Figure 2. The data for compact lattice loops was obtained using methods described in [10]. The trivial knotting probability for proteins is obtained by dividing the number of trivial knots found at a given length *N* by the total number of proteins with that length.

Considering only the protein data at 19 values of *N* for which nontrivial knots occur (abscissa less than 1), we see a clear downward trend of the trivial knotting probability. However, these represent only a very small fraction of the total number of protein data points (673). Moreover, the average number of protein chains with the same length *N* is very small (about seven chains per data point). Therefore, the dependence of the trivial knotting probability on the length, *P_trivial_*(*N*), is very weak. A linear fit to the protein data gives a slope of about −10^−5^. Using this small number, but remembering all the caveats just mentioned, we estimate a characteristic length of knotting of *N*_0_ ≈ 10^5^, where *P_trivial_*(*N*) ~ exp(−*N*/*N*_0_). This should be compared with the values estimated for random compact lattice loops as well as for noncompact unrestricted loops, where *N*_0_ ≈ 200 − 250 [10,19,20].

Thus, we conclude that knots in proteins are indeed orders of magnitude less frequent than a random polymer of comparable length, compactness, and flexibility would have it. Closer scrutiny of the subchain data, Figure 1, enables us to gain some insight into this lack of knots.

### Local Geometry

Returning to Figure 1, let us note that there are both similarities and important differences between the subchain scaling in proteins and lattice loops. The similarity, which we used above to make the comparison of knotting probabilities, is that both proteins and lattice loops exhibit random walk scaling 〈*R* ^2^(*ℓ*)〉 ~ *ℓ* over some range of scales. In both cases, this is a manifestation of the Flory theorem [14,21,22]. For proteins, random walk behavior consistent with the Flory theorem is observed for large subchains, starting at about 40 residues. The compact lattice loops also exhibit random walk behavior, which saturates earlier as the compact lattice loop becomes smaller (illustrated in Figure 1 for globular loop sizes 4 × 4 × 4, 6 × 6 × 6, and 8 × 8 × 8). But there are also important differences with regard to where and how 〈*R* ^2^(*ℓ*)〉 deviates from the Flory theorem.

For the subchain lengths below about ten, the protein data indicate that protein subchains are significantly more stretched than random; statistically they are almost straight. This deviation between the protein data and the dashed line (or with the data for compact lattice loops) for subchain lengths from 2–10 must be attributed to the presence of secondary structures in proteins. The plot also shows that the size of the secondary structures does not scale with that of the whole chain.

The most interesting part of the protein data lies in the region between 15 and 40 residues. The mean square end-to-end distance grows very slowly with subchain length in this region, i.e., 〈*R* ^2^(*ℓ*)〉 strongly saturates. To see more clearly where this happens, the “local exponent” 2ν = log [〈*R* ^2^(*ℓ*_2_)〉/〈*R* ^2^(*ℓ*_1_)〉]/log(*ℓ*_2_/*ℓ*_1_), basically the slope in the *log–log* plot computed from two adjacent data points, is plotted in the inset of Figure 1. Between about 15 and 40 residues, the “local exponent” is smaller than even a third: *ν* < 1/3. That means of course that protein chains on these scales statistically have a very strong tendency to fold back on themselves, or crumple. The minimum of ν is seen to occur for subchains of length near 25 residues, which agrees with the value of about 24 residues for the location of the plateau in the end-to-end distance versus length of subchains [14]. These results also agree with the study on the size distribution of closed loops in proteins (a closed loop is formed when two non-adjacent *α*-carbons come into close contact), where a value of 25–30 residues gives the optimal loop size [15–17]. Finally we note that the scale of 40 residues is smaller than the typical size of single-domain proteins, which is about 170 residues.

Intuitively, the “saturation” of the mean square end-to-end distance of protein subchains at these lengths should be expected to reduce the interpenetration of subchains relative to the situation in which the saturation is absent. In turn, we expect a low degree of interpenetration to lead to a suppression of knots. To measure the degree of interpenetration, we define the following quantity. Consider a subchain (inset of Figure 3) and take a sphere around its center of mass. The sphere's radius is equal to the gyration radius of the subchain. Count the number of units, excluding those belonging to this subchain, as well as the total number of units within this sphere. The ratio of the average of each of these two numbers over subchains with the same length is the degree of interpenetration plotted in Figure 3.

**Figure 3:**
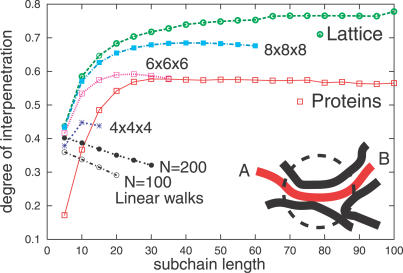
Degree of Interpenetration of Subchains Defined as follows: given a labeled subchain (say chain *AB* in the inset at the lower right), determine the fraction of the number of units (or residues) of the loop or protein enclosed within a sphere (dashed circle) that does not belong to the subchain. The radius of this enclosing sphere is equal to the gyration radius of the subchain. The degree of interpenetration is then defined as an average of this quantity over all subchains of the given length, taken within a single protein chain and from all other protein chains. As in the results for the mean square end-to-end distance, for each chain of length *N,* we consider subchains of length up to N^2/3^. The degree of interpenetration for proteins, lattices (average from five globular loop sizes from 4 × 4 × 4 to 12 × 12 × 12, and separately for 4 × 4 × 4, 6 × 6 × 6, and 8 × 8 × 8) and linear equilateral random walks of length *N* = 100 and *N* = 200 are shown.

We see that the degree of interpenetration for proteins is less than that of 6 × 6 × 6 loops. The degree of interpenetration for proteins is also less than that of 4 × 4 × 4 loops for subchain lengths up to about ten. For the short lengths, it should be less for proteins because of the smaller density. The degree of interpenetration for proteins saturates at about 30, which is also about where the mean square end-to-end distance saturates.

We also show the degree of interpenetration for linear equilateral random walks of the lengths typical for proteins, *N* = 100 and *N* = 200. The equal-length segments of the random walks are off-lattice and have zero thickness (i.e., no excluded volume). In this case, the degree of interpenetration is less than that of proteins for subchains longer than ten units. However, for random walks and in the swollen phase of polymers, it is known that the knots are localized or show up at scales much smaller than the whole chain [23,24] (the preferred size of trefoil-determining portions is about seven freely jointed segments [23].) Incidentally, a comparison of the degree of interpenetration for proteins, compact loops, and random walks also confirms that proteins are much more similar to compact loops than to random walks.

## Discussion

Thus, based on measurements of either end-to-end distance or degree of interpenetration, we see that native proteins are much more segregated on the intermediate scale up to about 40 residues than random compact conformations. It is obvious that the segregated character of a conformation is a very strong suppressor of knots. We can make the conclusion that the “Universe” of protein conformations is statistically consistent with the elimination of the vast majority of knots.

To discuss our findings, first of all we have to decide which question we want to ask. We do not understand what is the cause and what is the consequence: is knot suppression a byproduct of a certain mechanism selecting conformations on a purely local basis, such as, e.g., a certain type of crumpling, a certain local fractality, etc? Or is it the other way around, that local fractality and crumpling are the result of selection against knots?

Indeed, it seems natural to argue that native conformations with knots have been evolutionarily eliminated because they are not good for folding. Although it sounds plausible, not much solid support exists for this conjecture. At least for the lattice 36-mer it was possible to design a sequence with six sorts of monomers that was reliably folding into the native structure with a knot (unpublished data), and folding time was not dramatically longer than for other 36-mers with unknotted native states, under the same conditions. On the other hand, in recent simulation works [25–28] knots were seen as off-pathway folding intermediates for short peptides. This observation seems consistent with the idea that knots in general are impediments to folding.

Viewed at another angle, the rarity of nontrivial knots in proteins, well noted in several works [1–5] and confirmed here, presents a puzzle to the school of thought that protein dynamics is ergodic. Because the vast majority of long, collapsed chain structures are knotted, the relative absence of knotted proteins may indicate that not all conformations are being visited when a protein folds to its native state [1]. From this point of view, it is natural to conjecture further that the globular protein inherits the knot state of the denatured protein from which it is formed [2]. Such a view, although logically possible, seems difficult to reconcile with the fact of reliable folding over a (relatively) wide range of temperatures. Also, the recent observation of knotted off-pathway folding intermediates suggests that protein chains may, at least in some cases, visit knotted conformations.

The idea that crumpling, or folding back on itself, helps in suppressing knots is an old one [29]. In this old work, it was conjectured that the turns of *α*-helix or *β*-turns play the role of smallest scale crumples. Our present findings indicate quite the opposite, that in fact secondary structure leads to increased local ν index (up to about the scale of ten residues, see Figure 1), and only at the larger scale does the chain fold back on itself.

When this article was being revised for resubmission, [30] became available to us. There the authors claim that the (almost) complete repertoire of possible protein conformations can be reproduced based on a model that carefully takes into account only two factors, namely, overall chain compactness and hydrogen bonds. It would be interesting to look at this statement from the point of view suggested by us in this article. Indeed, if the model suggested in [30] reproduces the “protein universe,” its conformations should have few knots, if any. Although we do not know how to reconcile this with the observed knotted proteins, including some rather complex knots (see Figure 4 and [7]), more important would be the comparison of the statistics of knots in real proteins and in the model of [30]. In principle, one could speculate that while compactness strongly enhances the probabilities of knotted conformations [10,24], it is possible that the presence of hydrogen bonds, which is the indispensable and important additional feature of the model of [30], somehow suppresses knotting. (Similar consideration arises also in the context of the so-called tube theory, the latest versions of which include hydrogen bonding [31].) Possibly, the hydrogen bonds achieve this suppression by producing the hump in the plot of the subchain size similar to our Figure 1, or in other words, by affecting the local geometrical/fractal structure of the typical conformations.

**Figure 4:**
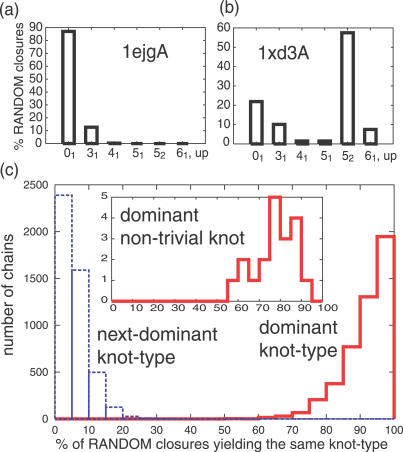
Dominance of Knot Types in the RANDOM Knot Closure (A) Percentage of the 1,000 RANDOM chain closures yielding the various knot types for the protein chain with ID 1ejgA and length *N* = 46. In this chain, the trivial knot or unknot (0_1_) dominates, while the trefoil knot (3_1_) is the next-dominant knot type. Both CENTER and DIRECT methods also predict a trivial knot. (B) Percentage of the 1,000 RANDOM chain closures yielding the various knot types for the protein chain with ID 1xd3A and length *N* = 229. In this chain, the knot 5_2_ dominates, while the trivial knot is the next-dominant knot type. The CENTER method also predicts a 5_2_ knot, while the DIRECT method detects a trivial knot. (C) Histogram of the percentage of RANDOM chain closures giving the dominant (solid steps) and next-dominant (dashed boxes) knot types within a single chain for all 4,716 protein chains. The inset shows the histogram for the percentage of closures giving the dominant knot type that is not a trivial knot.

To summarize, we have shown that native protein conformations have statistically much fewer knots than what random chance would imply (at least compared with a generic lattice model). We have also established a connection between the rarity of knots in native proteins and their local geometrical properties, such as subchain size and subchain interpenetration. It is tempting to hypothesize that any set of compact globular conformations will have few knots, or about as many as proteins do, provided it has the proper local geometrical peculiarities, including pieces having an extended configuration at small scales (mimicking secondary structures) and a compact or looped structure at intermediate scales (resembling motifs or small domains). To test this hypothesis, we need a better computationally tractable model of conformations, one capable of reproducing at least qualitatively the subchain behavior in Figure 1. Finally, although we can conclude that the present day “protein universe” largely avoids knots, we do not know whether knots have been removed through evolution because of their adverse effect on folding or function, or the suppression of knots came as a byproduct of some hitherto unidentified property of protein evolution.

## Materials and Methods

### Representative protein chains.

We extracted 4,716 protein chains from Protein Data Bank [32] (PDB) files available online. The PDB IDs, or code names, of the proteins can also be obtained online from the Parallel Protein Information Analysis system's Representative Protein Chains from PDB (PDB-REPRDB) [33]. PDB-REPRDB is a reorganized database of protein chains from PDB. Each group consists of chains similar to each other in terms of either sequence or structure. Each representative chain has the best quality in each chain group. The PDB IDs of the 4,716 representative protein chains can be obtained from a PDB-REPRDB sample table (dated 25 March 2005, based on PDB release 06 March 2005) with the following selection criteria: each representative is different from all other representatives in terms of a sequence similarity of *ID*% ≤ 30% and 3D structure similarity of *D_max_* ≥ 50 *Å*. (The sequence similarity (*ID*%) is the percentage of identical amino-acid residues between corresponding residue pairs in the two compared sequences. The 3D structure similarity (*D_max_*) is the maximum *α*-carbon distance between the corresponding residue pairs in the two compared structures.)

We verify that two well-known characteristics of proteins are shared by our sample. First of all we check that the radius of gyration (*R_g_*) of protein chains in our ensemble indeed scales with chain length (number of residues *N*) in a manner similar to that of compact polymers (i.e., *R_g_* ~ *N* ^1/3^) [14,34–38]. Moreover, the average bulk density of the proteins is approximately constant. We also measured the density of the residues enclosed by a sphere centered on the center of mass of a protein. The average density of the proteins is approximately constant at about (6*α* – carbons)/(10 *Å*)^3^ for sphere radii up to about the radius of gyration of the chain *R_g_*. As the sphere radius increases further, the density falls off to about half at a sphere radius of 1.5 *R_g_*.

We further address the issue of the predominant location of the terminals of the protein and show that they do tend to stay far away from the center of mass, an observation already noted in [2]. This result gives hope that the closure of proteins via an external loop can give an unambiguous account of the knot state. The distribution of the distance of each terminal to the center of mass (*R_T_*) is presented in Figure 5. The horizontal axis is scaled to the maximum residue distance from the center of mass (*R_max_*). The plot strongly suggests that the terminals prefer to be near the surface of the protein.

**Figure 5:**
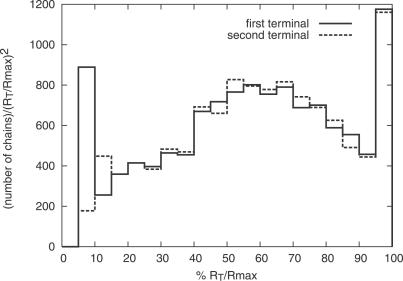
Distribution of the Distance of a Protein Terminal from the Center of Mass (*R_T_*) (*R_max_* is the distance of the residue farthest from the center of mass of the protein chain.) The distribution is divided by (*R_T_*/*R_max_*)^2^ to obtain a density and to take into account that a point chosen at random within a sphere is more likely to be found away from the center of the sphere.

We also mention some statistics regarding the secondary structure content (alpha helix or beta sheet) of the proteins. Most of the proteins in our sample contained at least one alpha helix and one beta sheet. Using the HELIX and SHEET entries in the PDB files, we found that the average fraction of residues in a chain participating in a helix is about 44%, while the average fraction of residues in a chain participating in a beta sheet is about 27%. The average length of a helix is about 11 residues, while the average length of a beta sheet strand is about five residues.

Although we take a protein chain to be the basic unit with which to perform our study of knots, it can be argued that it is a domain, representing a single globule, within the protein chain that can be compared statistically to the globule state of our model chains. Such domains are biologically significant because they are roughly defined as independent folding units [30]. As a step to address this issue, we use databases based on CATH, FSSP, and SCOP [39–42] to identify protein chains that consist of single domains. About one-third of the 4,716 protein chains in this study have been identified as single domains (CATH version 2.6.0 identifies 1,713 of the 4,716 chains as each comprising a single domain). The average length of these domains is about 170 residues.

### Chain closure methods.

The simplest way to complete a loop from an open linear chain is to connect the two terminals (first and last *α*-carbons) directly with a straight line. We call this method the DIRECT method, illustrated in Figure 6A.

**Figure 6:**
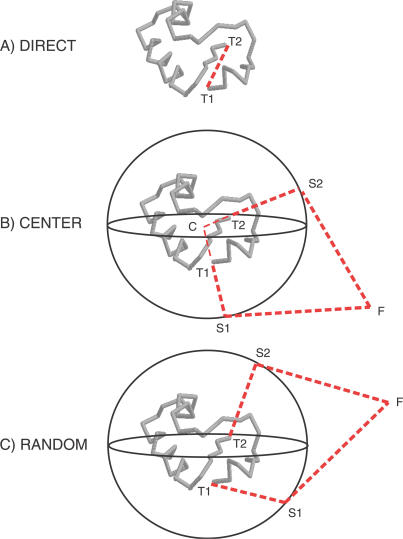
Illustration of the Three Chain Closure Methods The examples in these figures use the protein chain with PDB ID 1ejgA, rendered using Rasmol (R. Sayle). (A) DIRECT method. *T*_1_ and *T*_2_ refer to the terminals of the chain. We connect the terminals by the straight segment *T*_1_ – *T*_2_. (B) CENTER method. We enclose the entire chain in a sphere centered at *C,* the center of mass of the chain. We take straight lines starting at *C,* passing through the terminals *T*_1_ and *T*_2_, and intersecting the surface of the enclosing sphere at the points *S*_1_ and *S*_2_. *S*_1_ and *S*_2_ are connected to point *F,* located sufficiently far away outside the sphere, on the plane formed by *C, S*_1_, and *S*_2_. The closed loop is formed by the protein chain backbone complemented by the broken line *T*_1_ − *S*_1_ − *F* − *S*_2_ − *T*_2_. (C) RANDOM method. The points *S*_1_ and *S*_2_ are randomly positioned on the surface of the enclosing sphere whose center coincides with the center of mass of the chain. *S*_1_ and *S*_2_ are connected to point *F,* located sufficiently far away outside the sphere, on the plane formed by the center of mass, *S*_1_ and *S*_2_. A closed loop is again formed by the protein chain backbone complemented by the broken line *T*_1_ − *S*_1_ − *F* − *S*_2_ − *T*_2_.

In the CENTER method, (Figure 6B), the terminals are connected to the surface of a sphere enclosing the whole chain. The segments connecting the terminals to the sphere are extensions of the segments connecting the center of the sphere (coinciding with the center of mass of the chain) to the chain terminals. Because the terminals tend to stay near the surface of the protein, this method minimizes the portion of the chain that the connecting segments may pierce through. The two points on the sphere are then connected to a point a bit farther away from the center to complete the loop. Note that this method has a similar effect as holding the terminals fixed, then shrinking or smoothing the segments in between to find the knotted core [3–6,24]. (A beautiful version of this shrinking technique for closed loops is the Shrink-On-No-Overlaps method described in [43].) This shrinking or smoothing technique also shares similarities with various preprocessing schemes for reducing the length and number of crossings of a loop [8,10].

Finally, to reduce any bias in the chain closure, the two points on the sphere may be chosen randomly. We call this the RANDOM method, illustrated in Figure 6C. One can then compile the statistics of the knots formed by many random loop closures after generating several pairs of points. This method is similar to that used in [1,12,13]. In [12,13], the terminals are connected to a single point located randomly on a large sphere enclosing a chain.

The probabilistic definition of knots in proteins and other open chains, obtained from many random closures, is fundamentally more reliable than any deterministic method, e.g., those described in this section [1,12]. We shall see that in fact the knot type indicated by the CENTER method quite often agrees with the dominant knot type predicted by the RANDOM closure method. In this sense, the results of the RANDOM method provide more credence to the simpler CENTER method. In retrospect, this can be understood as due to the protein terminals tending to be at the periphery of the protein globule, and, therefore, connecting them to infinity is basically safe. It is also understandable then that the results of the DIRECT method, although not bad, are in significant disagreement with the RANDOM method.

### Knotting probabilities.

As in our studies with compact and unrestricted loops [10,19], we use the Alexander invariant (|Δ(−1)|) [44] and the Vassiliev invariants (*v*_2_, |*v*_3_|) [45] as signatures to identify the knot type of a chain for each closure method. We determine which chains have invariants corresponding to the knot types 0_1_ (trivial knot, unknot, or “no knot”), 3_1_ (trefoil), 4_1_ (figure-eight), 5_1_, and 5_2_. (In the standard nomenclature of knots, the first number indicates the number of minimal crossings in a projection of a knot. In this study, we do not distinguish between mirror images). Other more complicated knots with at least six crossings are lumped together, although the precise type of knot can be identified easily. The knot counts are listed in Table 1.

**Table 1:**
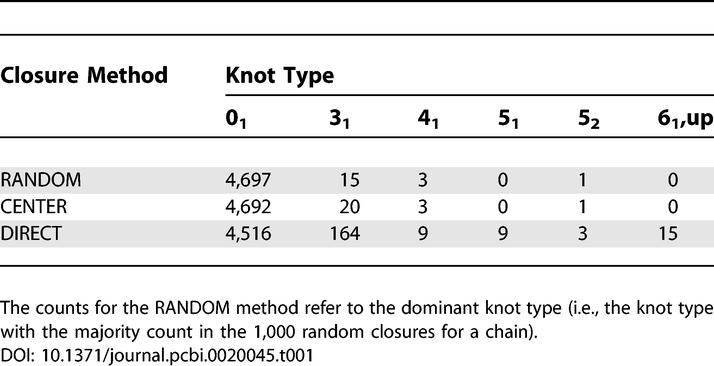
The Counts for the Different Knot Types Determined from 4,716 Protein Chains for the Three Closure Methods

There is no complete set of knot invariants that could determine the knot type exactly. For instance, the Alexander invariant and the Vassiliev invariants used in this work are guaranteed to identify a knot type unambiguously only for knots with nine crossings or fewer in the plane projection. To determine the accuracy of the use of knot invariants, we look at the number of crossings in the knot projections after the crossings were reduced with Reidemeister moves [10]. In the CENTER method, we find that in 4,598 out of the 4,716 chains, reduced crossings of nine or fewer are obtained, which means that the knot types are determined exactly by knot invariants for more than 97% of the chains (and we believe that false identifications for knots with more than nine crossings occur rarely). The average number of reduced crossings in the CENTER method is less than 1, which also implies that most of the knots in the protein chains are trivial knots. Similar results for the crossings can be found in the RANDOM and DIRECT methods.

In studying the statistics of the knots in the RANDOM closure method, we generate 1,000 pairs of points (*S*_1_,*S*_2_, see Figure 6C) randomly located on the surface of an enclosing sphere for each chain. Thus each chain yields 1,000 knots for analysis. Figure 4A and 4B gives examples of the knot probabilities generated by the RANDOM closures for two specific protein chains.

A single knot type is found to overwhelmingly dominate the RANDOM closures for each chain. For 4,021 chains out of the total number of 4,716 (85.3%), more than 85% of the knot closures for each chain yield the same knot type. In contrast, no more than 35% of the closures for any single chain yield the next-dominant knot type. The distributions of the percentage of RANDOM closures giving the dominant and next-dominant knot types are presented in Figure 4C. This “bimodality” in the distribution of knot types in the RANDOM closures for proteins is also present in the study of knots in linear random walks [12,13].

There is also close agreement between the dominant knot type determined with the RANDOM method and the knot types determined with the CENTER and DIRECT methods. The agreement is almost complete between the RANDOM and CENTER methods: every chain in 4,711 has the same knot type under these two methods (the CENTER method detects five additional trefoil knots). The agreement is significantly less between the RANDOM and DIRECT methods: every chain in 4,528 has the same knot type under these two methods. The significant number of extra knots detected in the DIRECT method is understandable in the light of the compactness and the terminals tending to stick out of the bulk: for the DIRECT method, the segment connecting the terminals pierces through more space occupied by the protein chain compared with the other two methods.

As we already mentioned, to compare proteins to lattice loops in terms of their knotting probabilities, we need to be able to bring these two systems into comparable scale. The way to do that is well understood in polymer physics. It should be based on the study of subchains.

### Subchains.

By subchain in this context we understand just a part of the polymer chain: a subchain of the length *ℓ* may be from any monomer number *i* to monomer *i* + *ℓ*. The study of subchains for the single polymer globule plays the same role as looking at a labeled polymer in the macroscopic melt [21]: the subchain end-to-end distance squared, 〈*R* ^2^(*ℓ*)〉, averaged over *i* in any given conformation (“sliding window average”) and probed as a function of *ℓ*, reveals the scale-dependent conformational properties. The reason why subchains are relevant to understanding the knotting probabilities can be understood from this simple limit: suppose that the polymer chain is completely straight. Then, every subchain would have its end-to-end distance scaling as *ℓ*, and there will be no knots whatsoever.

Figure 1 presents data for the mean square end-to-end distance of subchains of proteins and compact lattice loops plotted against the subchain length. For each chain of length *N*, we considered (and averaged over) the subchains of length up to *N* ^2/3^. The data for compact lattice loops come from random loops with lengths along one dimension of *L* = 4,6,8,10,12 (*N* = *L* ^3^) and with 100 samples for each *L*. The data for proteins is similar to that obtained in [14]. We emphasize that a similar curve is also obtained (bearing the characteristic “hump” displayed in Figure 1) whether we consider “small” proteins (e.g., less than 200 residues), “large” proteins (e.g., greater than 200 residues), or single-domain protein chains.

The subchain length for both proteins and lattice loops is measured in the number of monomers (less 1). As to the end-to-end distance, it is measured naturally in lattice constants for the lattice system, while for proteins the corresponding measurement unit was adjusted to achieve a close match between the data for proteins and lattice loops in Figure 1. We found that a close match is achieved if *R* ^2^(*ℓ*), originally measured in (angstroms)^2^ for proteins, is divided by (3.8)^2^; this is what is shown in Figure 1. Notice that since Figure 1 represents the *log–log* plot, this scale factor can only move the curve up or down. The dashed line in the plot corresponds to the random walk behavior 〈*R* ^2^(*ℓ*)〉 = *ℓ,* which implies a Kuhn length of 1 for the lattice system or about 3.8 *Å* for proteins (3.8 *Å* is also the typical distance between adjacent *α-*carbons along the chain).

At first glance, this value is in contradiction with the known persistence length of coil-like protein chains, which is usually believed to be about 7 *Å*. This follows from both theoretical calculations of the flexibility of polypeptide chains [46] and from recent systematic studies of denatured proteins [47]. In fact there is no contradiction, because in our statistical study the conformations of subchains are buried within quite dense globules; in other words, the subchains are surrounded by the protein medium, while in the coil-like denatured state of the protein, the monomers are mostly surrounded by solvent. On a more rigorous level, one can say that the Flory theorem [21] implies that polymers should have Gaussian statistics in the melt (i.e., in the environment of similar polymers), but the theorem does not say what the persistence length should be—it need not be the same as in the denatured coil.

### Protein knots.

In this section we list the code names of the 19 protein chains determined with the RANDOM closure method as each being knotted. About one-third of these chains are single domains according to CATH version 2.6.0. A visual inspection with the aid of Rasmol (R. Sayle) reveals that some of the chains have breaks or discontinuities.

The proteins with trefoil knots (3_1_) are: 1lugA, 1v2xA, 1o6dA, 1mxiA, 1ualA, 1vhyA, 1t0hB, 1js1X, 1k3rB, 1x7oA, 1p7lA, 1gz0E, 1vhkD, 1gkuB, 1xi4C.

The proteins with figure-eight knots (4_1_) are: 1qmgA, 1u2zC, 1m72B.

The protein with the knot 5_2_: 1xd3A.

(The first four characters in the code is the PDB ID, while the fifth character identifies the chain within the protein.)

After this work was submitted, we were made aware of [7], which also identifies the 19 proteins as being knotted and gives further details on the molecular biology of these and other knotted proteins. In particular, [7] also identifies the knot 5_2_ in the protein 1xd3 (ubiquitin hydrolase UCH-L3), and also suggests that the occurrence of this knot might be related to the role of the enzyme in protein degradation.

## Abbreviations

PDB: Protein Data Bank
